# Interprofessional survey on knowledge and attitudes of midwives regarding oral health, in France

**DOI:** 10.18332/ejm/172881

**Published:** 2023-12-01

**Authors:** Abid Bossouf, Céline Sabourin, Florent Fuchs, Nicolas Giraudeau, Camille Inquimbert

**Affiliations:** 1 Department of Public Health, Faculty of Dental Medicine, University of Montpellier, Montpellier, France; 2 Center for Latin European Political Studies, UMR 5112, CNRS, University of Montpellier, Montpellier, France; 3 Team of Criminal Law and Forensic Sciences of Montpellier, UR-UM212, University of Montpellier, Montpellier, France; 4 Department of Obstetrics and Gynecology, Montpellier University Hospital Center, Montpellier, France; 5 National Institute of Health and Medical Research, Centre for Epidemiology and Population Health, Reproduction and child development, Paris, France; 6 Desbrest Institute of Epidemiology and Public Health, University of Montpellier, Montpellier, France

**Keywords:** knowledge, pregnancy, midwives, attitude, literacy, oral health

## Abstract

**INTRODUCTION:**

Oral health is essential for psychosocial well-being and general health. For expectant mothers, pregnancy increases the risk of oral diseases and has a subsequent impact on the oral health of a child once born. Midwives are in charge of pregnancy monitoring, childbirth and newborns’ first days of life. They could have an important role in prevention. However, limited studies evaluating the knowledge, attitudes and practices on oral health among midwives have been conducted in Europe.

**METHODS:**

We performed a cross-sectional study using a self-administered questionnaire. Two local midwifery associations sent out the questionnaire by email and social media networks to all registered midwives and practicing in the department of Herault (n=613), between April and May 2022. Statistical analyses on quantitative data and descriptive analyses of qualitative free-text responses were performed.

**RESULTS:**

In total, 167 midwives were included. We found a lack of knowledge on many oral health topics and this was stated as the main reason that only 29% of midwives provided oral health information to their patients. Only 30% of the midwives had a training module on oral health during their initial training, and less than half of them considered the training adequate. To improve their lack of knowledge, participants expressed a preference for digital communication methods for themselves; however, they favored in-person interaction for public interventions.

**CONCLUSIONS:**

This study showed a lack of training and knowledge about oral health among midwives and a lack of oral health discussion with expectant mothers who are a high-risk population for oral diseases.

## INTRODUCTION

Oral health is an essential part of the psychosocial wellbeing and general health of a person^1^. The World Health Organization defines oral health as: ‘the state of the mouth, teeth and orofacial structures that enables individuals to perform essential functions, such as eating, breathing and speaking, and encompasses psychosocial dimensions, such as self-confidence, well-being and the ability to socialize and work without pain, discomfort and embarrassment’; and that it ‘varies over the life course from early life to old age, is integral to general health and supports individuals in participating in society and achieving their potential’^2^.

High prevalence of oral health diseases is a public health concern. Worldwide, 3.5 billion people are affected by oral diseases and over 514 million children suffer from caries of primary teeth^2^. In France, in 2019, almost 30% of children aged 1–9 years had untreated caries of deciduous teeth^3^. However, most oral health problems are avoidable if treated at an early stage and prevention is key in the fight against oral disease.

Pregnant women are a particularly high-risk population for oral health diseases. Multiple studies have shown a relationship between oral health during pregnancy and subsequent complications^4-6^. Yousefi et al.^4^ showed that the physiological changes involved during pregnancy can increase the risk of cavities, and Dye et al.^5,6^ found that untreated periodontal disease has a negative effect on pregnancy and on newborn health (such as premature birth, fetal growth restriction, or pre-eclampsia). Therefore, the mother’s oral health is related to the future child’s oral health^5,6^.

Among healthcare professionals, midwives have a unique role to play since they are in charge of pregnancy monitoring, childbirth and newborns’ first day of life^7^. It could therefore be said that midwives should be equipped to act and promote oral health. Even though studies evaluating oral health knowledge and attitudes among midwives have mostly been conducted in the USA^8,9^ and Australia^10,11^, limited studies have been carried out in Europe^12-14^. The objective of this study was to assess the oral health knowledge and attitudes of midwives in the department of Herault in France.

## METHODS

### Study design

An observational, descriptive, cross-sectional, multi-center study was conducted using a self-administered questionnaire. This pilot study was part of a bigger project called Montpellier Santé Orale and similar studies have been conducted for nurses, speech therapists, physiotherapists, pharmacists, pediatricians, and medical general practitioners (GPs).

### Sample and setting

Two local midwifery associations sent out the questionnaire by email and social media networks to all registered midwives and practicing in the department of Herault (n=613), between April and May 2022. A purposive sampling technique was used and informed consent was obtained from all participants.

The protocol and study design were approved by ethics and regulatory agencies and were implemented in accordance with provisions of the Declaration of Helsinki. The appropriate Committee (Montpellier University Hospital Institutional Review Board, Montpellier, France) approved the protocol (IRB 2022_10_202201250).

Participants had to sign the inform consent. The informed consent form contained the name and affiliation of the investigator, a plain language description of the study, and the ethics committee approval.

### Data collection

The questionnaire (Supplementary file) was developed as part of a collaborative multidisciplinary project and was based on the one conducted in Australia^10^. It was distributed online using Google Forms.

The first part of our questionnaire was relevant to all healthcare professionals (including midwives, nurses, speech therapists, physiotherapists, pharmacists, pediatricians, and medical general practitioners), whereas the second part was specifically given to midwives.

The questionnaire integrated both quantitative and qualitative elements. It was divided into four sections. There were three question types: five open-ended questions, eight yes or no questions, and five multiple choice questions. The first section collected information about the survey respondent’s background and training. The level of teaching was based on the declarative values and perceptions of the participants. The second section (14 questions) assessed their understanding of the relationship between oral health and general health. The third section (17 questions) assessed the respondent’s understanding of prevention, hygiene habits, and the role of food/drink in oral health. Lastly, the fourth section collected information about the respondent’s knowledge of the relationship between pregnancy and oral health, approach during consultations and oral health prevention strategies preferences for themselves as well as with the general population.

### Data analysis

Incomplete questionnaires were excluded from the study. Microsoft Excel was used to perform the statistical analysis and the results are presented in percentages. Participants with missing data were excluded from any analyses involving that data. Data regarding the oral health knowledge and attitudes of midwives was summarized using descriptive statistics including frequencies, means and standard deviations. The Mann-Whitney U test was used to check whether there was a difference in correct responses, depending on the initial training. The significance threshold was set at 5%.

## RESULTS

### Participants

The total sample comprised 167 midwives. The response rate was 31.5% (n=193) but we excluded 26 questionnaires: midwives not practicing in the department of Herault, or incomplete questionnaires.

Most participants were female (98.8%), aged 24–62 years, with a mean of 37.3 ± 9.85 years. Most (53.9%) participants reported working in a hospital setting, 34.7% in private practice and 16.2% in private clinic, with some overlap between the groups since many participants had multiple professional practices. A range of levels of experience was reported, with participants stating that they had practiced midwifery between 1 to 41 years (mean=13.1 years). More than 75.0%, however, stated that they practiced for <20 years.

Most respondents (62.3%) completed their training in the department of Herault. Overall, only 30% of the participants said that they had taken an oral health module during their training, and only 13.2% expressed that they received adequate training.

**Figure 1:**
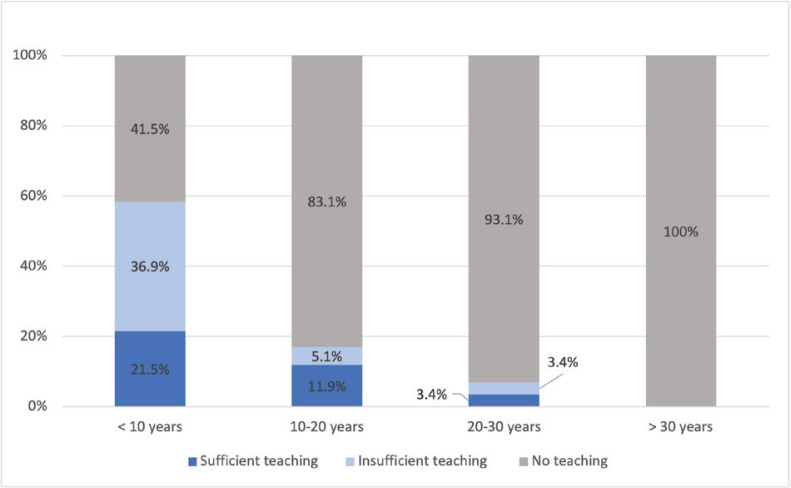
Proportion of midwives (n=167) who received training on oral health according to the number of years since their midwifery qualification, in the department of Herault in France (2022). The level of teaching was based on the declarative values and perceptions of the participants

We found that the midwives who had received their credentials more recently were more likely to have received training on oral health (Figure 1). Of the 13 participants (7.8%) who had received training on oral health since their qualification, all had qualified in or before 2007.

### Knowledge

For the questions on the relationship between oral health and general health, we received correct answers for 75.8% of the time (Table 1). The least correctly answered question was on whether oral bacteria can reach the amniotic fluid and lead to infections in expectant mothers (47.9% of participants answered this correctly), whereas the most correctly answered question was about tobacco use and that it increases the risk of upper respiratory tract cancer (98.2% of participants answered this correctly).

**Table 1:** Knowledge and attitudes of midwives on the relationship between oral health and general health, Herault, France, 2022 (N=167)

*Statement*	*True/false*	*Correct responses*	
		*n*	*%*
Studies show that there is little correlation between oral health and general health	False	154	92.2
Oral bacteria can spread through the organism via the blood and airways	True	145	86.8
Oral bacteria can reach the amniotic fluid and lead to infections in pregnant women	True	80	47.9
There is a bidirectional correlation between periodontal disease and diabetic control	True	92	55.1
Tobacco use increases the risk of upper respiratory tract cancer	True	164	98.2
There is an oral prevention examination 100% covered by Health Insurance from the 4th month of pregnancy	True	150	89.7
Dental care during pregnancy is limited to emergency care	False	149	89.2
The M’T Dents (prevention examination) concerns children from the age of 3 years	True	103	61.7
Oral bacteria can increase the risk of cardiovascular disease	True	104	62.3
Heredity can play a role in certain pathologies, but not for oral pathologies	False	154	92.2
The premature loss of baby teeth has no effect on permanent dentition	False	156	93.4
Respiratory problems in children can affect facial growth	True	95	56.9
The position of the tongue is an essential growth factor in skull development	True	113	67.7
Good oral health reduces the risk of lung disease	True	115	68.9
**Total correct responses**			**75.8**

Regarding the knowledge of midwives on food and drinks and their impact on caries prevention for their patients, we received correct responses for 74.7% of the time. The question that was correctly answered by all 167 respondents was on the products used by dentists for dental whitening and whether they are similar to those found over the counter (Table 2). The two least correctly answered questions were about the reimbursement by the national health insurance (*Assurance Maladie*) and whether children aged <6 years could use fluoridated toothpaste.

**Table 2:** Knowledge of midwives on food and drinks and their impact on caries prevention, Herault, France, 2022 (N=167)

*Statement*	*True/false*	*Correct responses*	
		*n*	*%*
Brushing your teeth in the morning and at noon is equivalent to brushing your teeth in the morning and evening	False	165	98.8
An electric toothbrush is always more effective than a manual toothbrush, even if you use a good brushing technique	False	90	53.9
Toothbrushes with soft, medium or hard bristles are equally effective as long as the correct brushing technique is used	False	94	56.3
The number of bristles on a toothbrush influences the quality of brushing	True	81	48.5
A toothbrush with hard bristles can cause irreversible damage to the gum tissues as well as to the dental tissues	True	119	71.3
It is necessary to clean the interdental spaces with dental floss or interdental brushes daily in addition to the brushing	True	117	70.1
Mouth cleaning is no longer useful when you have no more teeth	False	166	99.4
Fluoride is a natural mineral that can be found in tap water, tea or some fish	True	90	53.9
For children under 6, toothpaste should not be fluoridated	False	83	49.7
Drinking sodas too often causes dental erosion	True	161	94.4
The products used by the dentist for dental whitening are similar to those found over the counter	False	167	100.0
The cleaning of dental prostheses (removable) can be done with a manual or electric toothbrush	False	97	58.1
It is not necessary to clean baby teeth as soon as they appear	False	160	95.8
Intake of sugary/acidic foods or drinks throughout the day increases the risk of developing caries	True	156	93.4
A 4-year-old child can has cavities because of drinking milk from a bottle (prolonged use of the bottle during the day or when falling asleep)	True	144	86.2
Risk of caries decreases when the quantity of saliva decreases	False	149	89.2
**Total correct responses**			**74.7**

The least correctly answered questions were on those specifically regarding oral health during pregnancy (Table 3). These questions were only answered correctly 67.4% of the time. The least correctly answered question was whether maternal oral health status is related to child oral health status (27.5% of participants answered this correctly).

**Table 3:** Knowledge of midwives regarding oral health during pregnancy, Herault, France, 2022 (N=167)

*Statement*	*True/false*	*Correct responses*	
		*n*	*%*
An oral pathology (periodontitis; caries, abscess) can cause a gynecological/obstetrics complication	True	146	87.4
Pregnant women are more susceptible to the development of periodontal disease than the average person	True	156	93.4
Maternal oral health status is related to child oral health status	True	46	27.5
**Total correct responses**			**69.4**

### Training

We did not find a correlation between the percentage of correct responses and completion of training on oral health during initial training (Mann-Whitney test, p=0.4). The percentage of correct answers was 71.3% for participants without oral health module during their training and 75.8% for participants with the module.

There was also no difference found between the percentage of correct responses and having completed training considered ‘adequate’ (Mann-Whitney test, p=0.7). The percentage of correct answers was 76.3% for participants declaring their oral health module insufficient and 75.2% for those declaring the module sufficient.

Many participants expressed the feeling that they had not received adequate training on oral health during their training. One midwife who graduated in 2015 stated: *‘During my training, we vaguely learned that there could be risks associated with the mother's oral health, but no more detail than that’*. Another participant who graduated their training in 2014 specified: *‘with this questionnaire, we realize how much we don't know about the subject’*.

Moreover, 70.1% reported to have never received an oral health module during their initial training; and for those who received it, 65.2% reported that it was insufficient. To remedy this, participants provided suggestions: 1) to have interdisciplinary exchanges within their initial training and beyond, 2) to create a manual for midwives containing key information, and 3) create training courses for all healthcare professionals.

### Attitudes

Most participants (70.7%) reported that they do not provide information about oral health during their consultations and that it is primarily due to a lack of knowledge on the subject (41.9%). Additionally, 76.6% of respondents stated that no printed information about oral health during pregnancy was provided to the expectant mothers that they assisted.

In the free text responses, participants mentioned that some dentists refused to see or treat pregnant patients.

### Prevention

The ways in which information on oral health could be taught to midwives and the general population varied (Table 4). Our results showed that midwives had a preference for multidisciplinary meetings (31.7%), followed by online training (21%), digital platform (20.4%), video clips (16.2%), books/magazines (4.2%), text messages (3.5%) and continuous training/university diploma (3%). In contrast, for educating the general population in oral health, the midwives favored preventive action (43.1%) followed by video clips (27.5%), distribution of flyers (13.8%), text messages (9.6%), digital platform (4.2%) and books/magazines (1.8%).

**Table 4:** Oral health prevention strategies for midwives and the general population, Hérault, France, 2022 (N=167)

*Actions for distributing information on oral health*	*Midwives %*	*General population %*
Preventative action	-	43.1
Multidisciplinary meetings	31.7	-
Online training	21.0	-
Digital platform	20.4	4.2
Video clips	16.2	27.5
Flyers	-	13.8
Text messages	3.5	9.6
Books/magazines	4.2	1.8
Continuous training/university diploma	3.0	-

Many comments referred to the need to improve prevention campaigns for the general population. One participant suggested raising awareness among politicians, while another suggested placing posters in waiting rooms. Some participants mentioned a lack of clarity in the information given by the French health insurance system about dental examinations from the fourth month of pregnancy.

## DISCUSSION

This study revealed that few midwives, in the department of Herault region, received specific training on oral health, and that there were gaps in their knowledge on oral health. We found similar results on the lack of education on oral health^8-14^. This may suggest that the lack of education and training on oral health for midwives is not isolated in Herault alone. It is also worth noting that we observed that the midwives who had received their credentials more recently had more often received training on oral health, which suggests that there has been recognition of the importance of training midwives about this subject^15^. However, no significance was found in the number of correct responses between those who had or had not received training. This suggests that even when training is provided, it may be inadequate and corresponds with the midwives’ own assessment in which less than half of those who had received training believed it to be adequate.

Regarding the participant characteristics, our findings were similar to the national statistics of midwives provided by the French government^16^. In 2021, France had 23400 midwives with an average age of 41 years for women and 36 years for men. Moreover, it was reported that 59% of midwives are employees and hospital workers, 34% of midwives are self-employed, 31% of midwives are in mixed practice, and 7% of midwives are classified as other.

A good understanding of the relationship between oral health and general health was stated among the participants. However, there were gaps in knowledge. Only 55.1% were aware of the bidirectional relationship between gum disease and diabetes. More generally, this concept seems to be poorly understood within the field. A study of French obstetrician-gynecologists (OB-GYNs) found that only 42.1% considered diabetes to be a risk factor in gum disease^17^. Similarly, prevention of oral health problems in relation to food and drink was observed. Midwives showed a good overall understanding; however, there were gaps in their knowledge.

Only 27.5% of participants from our present study knew that maternal oral health is related to the child’s oral health. This is very low compared to the literature, in which 86.2% of midwives agreed with the same statement in the USA as did 62.1% of midwives in Australia^8,10^. In future work, it could be worth considering the different training methods used and whether they could be applicable in France. For example, the New York State Department of Health edited a decisional tree to guide prenatal care providers and identify patients who require oral health^18^.

Although, integrating oral health into the midwifery curricula is still an issue, interprofessional training could be the key to strengthening the link between dentists and other healthcare providers. The objective would be to increase medical knowledge and therefore be more effective on giving holistic care, including oral care.

In 2010, the French health authority (*Haute Autorité de Santé*, HAS) stated that healthcare professionals working with expecting parents should receive training to give oral health advice relevant to parents and the child. The HAS also recommended that the subject of oral health should be discussed during midwifery consultations in the fourth month of pregnancy which matched with the knowledge of some participants free-text responses in our findings^19^.

In two previous research works, 80% of midwives consider themselves to be particularly well placed to discuss oral health prevention with the expectant mothers they cared for and that midwives have a key role to play^7,14^.

In our study, less than a third of the participants provided information about oral health to their patients, although it should be noted that for some, notably those working in emergency obstetric care, this question did not seem relevant. This was similar to a French study conducted on OB/GYNs which reported that only 26.3% of them discussed oral health with their pregnant patients^17^. This lack of willingness to broach the subject with patients could be due to a lack of knowledge. Nearly half (41.9%) of our participants stated this as their reason, and more than two-thirds of the participants in the Australian study felt that they did not have enough knowledge to give oral health advice^10^. Additionally, in a recent study of midwives in Brittany, France, 88.8% reported ‘insufficient’ or ‘non-existent’ knowledge about the link between oral health and pregnancy. Most (85.4%) addressed the topic of oral health ‘never’ or ‘sometimes’. In line with the results of our study, very few of these midwives had received training on oral health during their initial studies, and the vast majority reported an interest in receiving more training^20^. These results suggest that our observations are part of a wider trend throughout France.

Together, these results highlight that if the HAS recommendations are to be met, a significant improvement in oral health education and confidence-building among midwives need to be achieved. In addition to improving oral health education during midwifery training programs, the lack of knowledge, revealed in our study, highlights the possible need of improving awareness among already practicing midwives and the general population.

In terms of communication with healthcare professionals, participants showed a preference for the creation of a digital resource platform. A well-designed, easy-to-use interface could offer helpful resources for midwives and other healthcare professionals. The midwives we surveyed were also interested in multidisciplinary events that have already been shown to improve interdisciplinary understanding and communication^21^. The third most popular option was online training. An Australian study has shown the effectiveness of a self-directed online training course for midwives in that it found they helped midwives to integrate oral health promotion into their clinical practice^22^.

To confirm the interest of the use of digital technology to reach healthcare professionals, it may be worth noting that the WHO launched the mOralHealth Programme and the Handbook on ‘Mobile technologies for oral health: An implementation guide’ in 2021^23^. The program proposes a specific module on mOralHealth training to improve knowledge and skills of frontline health workers on oral health including midwives.

A systematic review, conducted in 2018, demonstrated the effectiveness of educational programs about oral health for the general population^24^. The most popular method among the midwives we surveyed was in-person interaction followed by video clips. The use of the email and social media has also been shown to be effective in communicating oral health information to the general population particularly in the form of training courses^25^. This should be taken into consideration when thinking about prevention campaigns. Text messages were less popular among our study population, but a study conducted in Iran, showed them to be an effective way of improving knowledge and attitude among pregnant women^26^. Similarly, the creation of a digital platform was not popular in our study, but has been shown to have a positive effect on knowledge and attitudes^27^. This raises the possibility that the methods preferred by the midwives are not necessarily the most effective in practice. The relative effectiveness of different communication strategies is therefore an important area for future research. It would be interesting to compare our results to the results obtained using the same base questionnaire with different healthcare professionals. This could reveal ways in which they could collaborate on informational content and/or prevention campaigns, for example, shared digital platforms or training courses.

### Limitations

Regarding the limitations, it was a pilot study with a small participant number and the use of the email. Also, email and social media networks to disseminate the questionnaire may have led to a potential selection bias towards a younger population. Additionally, the voluntary nature of the study means that the respondents were likely to be more interested in the topic compared to their colleagues who did not respond. It should also be noted that the structure of the questionnaire (providing statements to be marked true/false by the respondent) may have triggered some participants’ subject-specific memories that might not have otherwise been recalled.

## CONCLUSIONS

Despite increased attention on oral health education during midwife training, our study shows that midwives lack the required knowledge to fulfil this role. Systematic inclusion of an oral health curriculum in initial training could help to bridge this gap as could awareness and prevention campaigns for both midwives and for the general population. Future research should assess the most effective methods to employ.

## Data Availability

All anonymized data are available from the corresponding author upon reasonable request.
