# Supplementary material for: Interprofessional survey on knowledge and attitudes of midwives regarding oral health, in France
Source: Eur J Midwifery. 2023 Dec 1;7:37. doi: 10.18332/ejm/172881 (PMC10690821; doi:10.18332/ejm/172881)
Supplement: Supplementary file 1 [file EJM-7-37-s1.pdf]

Supplementary Material 1: Midwives Questionnaire (Part 2)

Questionnaire: Midwives' knowledge of oral health
**Question 1: How old are you?**<br><br>______________
**Question 2: Gender**<br><br>○ Female<br>○ Male
**Question 3: In what year did you qualify?**<br><br>______________
**Question 4: Where did you complete your qualification? (postcode)**<br><br>______________
**Question 5: Where are you currently practicing? (postcode)**<br><br>______________
**Question 6: In what setting do you currently work?**<br><br>○ Hospital<br>○ Clinic<br>○ Private practice

- Other: ______________

Question 7: Did you receive specific training in oral health during your qualification?

- Yes
- No

Question 8: If yes, do you believe the training was sufficient?

- Yes
- No

Question 9: Have you received any training on oral health since you qualified?

- Yes
- No

Question 10: Of the following statements related to oral/general health, please select those you agree with:

1. Studies show that there is little correlation between oral health and general health
2. Oral bacteria can spread through the organism via the blood and airways
3. Oral bacteria can reach the amniotic fluid and lead to infections in pregnant women
4. There is a bidirectional correlation between periodontal disease and diabetic stability
5. Smoking increases the risk of cancer in the upper respiratory tract
6. The French social security system fully refunds a dental consultation from the 4th month of pregnancy
7. Dental care during pregnancy is limited to emergency care
8. The M’T dents program (oral health prevention) is available to children from 3 years old.
9. Oral bacteria can increase the risk of cardiovascular disease
10. Genetics can play a role in some pathologies, but not in oral pathologies
11. Early loss of milk teeth has no effect on adult teeth.
12. Respiratory problems in children can have an effect on facial development.
13. Tongue position is a key factor in skull development.

14. Good oral health reduces the risk of lung disease

- Agree
- Disagree

Question 11: Of the following statements related to prevention and food/drink, please select those you agree with:

1. Brushing one’s teeth in the morning and at midday is the same as brushing one’s teeth in the morning and the evening.
2. An electric toothbrush is always more effective than a manual toothbrush, even with a good toothbrushing technique.
3. Toothbrushes with soft, medium or hard bristles are equally effective if used with a good toothbrushing technique.
4. The number of bristles on a toothbrush affects the quality of brushing.
5. A hard-bristled toothbrush can cause irreversible damage to gum and tooth tissue.
6. It is essential to clean the interdental spaces daily with floss or interdental brushes in addition to toothbrushing.
7. Toothbrushing/cleaning the mouth is no longer useful when the person has no teeth left.
8. Fluorine is a natural mineral found in tap water, tea, and some fish.
9. Children under 6 years old should use fluoride-free toothpaste.
10. Drinking fizzy drinks too often causes tooth erosion.
11. Products used by dentists for tooth whitening are similar to those available off the shelf.
12. The French social security reimburses two visits annually.
13. Removable dental appliances are cleaned using either a manual or electric toothbrush.
14. It is not necessary to brush milk teeth as soon as they appear.
15. Eating/drinking sugary or acidic food/drink throughout the day increases the risk of developing cavities.
16. A 4-year-old child can acquire cavities from drinking milk from a bottle (prolonged use of a bottle during the day or while going to sleep)
17. The risk of cavities decreases as the amount of saliva decreases.

- Agree
- Disagree

**Question 12: Do you agree with the following statement:** *"Dental disease (gum disease, cavities, infections) can cause gynecological/obstetrical complications (premature delivery, hypotrophic newborn, pre-eclampsia)"*<br><br>o Yes<br>o No
**Question 13: Select the statements you agree with**<br><br>o Pregnancy increases the risk of periodontal disease<br>o Maternal oral health is linked to the child's oral health<br>o Ankyloglossia (tongue tie) affects facial growth
**Question 14a: Do you provide information on dental hygiene during antenatal care?**<br><br>o Yes<br>o No
**Question 14b: If "no", why not?**<br><br>o Lack of time<br>o Lack of knowledge<br>o N/A: I already provide information<br>o I don't know
**Question 15: What kind of intervention do you think would be helpful for you as a healthcare professional? (by order of preference)**<br><br>o Video clips<br>o Oral health diploma<br>o Multidisciplinary evening event<br>o SMS messages<br>o Digital platform<br>o Book/magazine<br>o Online training

**Question 16: What kind of intervention do you think would be helpful for the general population? (by order of preference)**<br><br>○ Actions in the field<br>○ SMS messages<br>○ Distribution of flyers<br>○ Digital platform<br>○ Book/magazine<br>○ Video clips
**Question 17: Do you have any comments/suggestions/ideas?**<br><br>______________________
Thank you for your participation

© 2023 Bossouf A. et al.
